# Effects of 2 modes of positive pressure ventilation on respiratory mechanics and gas exchange in foals

**DOI:** 10.1111/jvim.16651

**Published:** 2023-04-13

**Authors:** Sharanne L. Raidal, Mel Catanchin, Muriel Sacks, Ann Carstens, Chris Quinn, Martina Mosing

**Affiliations:** ^1^ School of Animal, Environmental and Veterinary Sciences Charles Sturt University Wagga Wagga New South Wales Australia; ^2^ School of Veterinary Medicine, College of Science, Health, Engineering and Education Murdoch University Murdoch 6150, Western Australia Australia

**Keywords:** bi‐level positive airway pressure (bi‐PAP), computed tomography, continuous positive airway pressure (CPAP), equine critical care, equine respiratory physiology, neonatology, noninvasive ventilation (NIV), pressure support ventilation (PSV), respiratory insufficiency, respiratory support

## Abstract

**Background:**

Continuous positive airway pressure (CPAP) and pressure support ventilation (PSV) can improve respiratory mechanics and gas exchange, but different airway pressures have not been compared in foals.

**Hypothesis/Objectives:**

Assess the effect of different airway pressures during CPAP and PSV have on respiratory function in healthy foals with pharmacologically induced respiratory insufficiency. We hypothesized that increased airway pressures would improve respiratory mechanics and increased positive end‐expiratory pressure (PEEP) would be associated with hypercapnia.

**Animals:**

Six healthy foals from a university teaching herd.

**Methods:**

A prospective, 2‐phase, 2‐treatment, randomized cross‐over study design was used to evaluate sequential interventions in sedated foals using 2 protocols (CPAP and PSV). Outcome measures included arterial blood gases, spirometry, volumetric capnography, lung volume and aeration assessed using computed tomography (CT).

**Results:**

Sedation and dorsal recumbency were associated with significant reductions in arterial oxygen pressure (PaO_2_), respiratory rate, and tidal volume. Continuous positive airway pressure was associated with improved PaO_2_, without concurrent hypercapnia. Volumetric capnography identified improved ventilation:perfusion (V/Q) matching and increased carbon dioxide elimination during ventilation, and spirometry identified decreased respiratory rate and increased tidal volume. Peak inspiratory pressure was moderately associated with PaO_2_ and lung volume. Improved pulmonary aeration was evident in CT images, and lung volume was increased, particularly during CPAP.

**Conclusions and Clinical Importance:**

Both CPAP and PSV improved lung mechanics and gas exchange in healthy foals with induced respiratory insufficiency.

**Abbreviations**
ABG: arterial blood gas
biPAP: bi‐level positive airway pressure
CO_2_
: carbon dioxide
CPAP: continuous positive airway pressure
CSU: Charles Sturt University
CT: computed tomography
ECG: electrocardiogram
ETCO_2_
: end‐tidal CO_2_
FiO_2_
: fractional inspired O_2_ concentration
HR: heart rate (bpm)
Index_Eng_
: Enghoff's index
MAP: mean arterial pressure (mm Hg)
MV: minute ventilation
MVe: expiratory minute ventilation
NIV: noninvasive ventilation
NTT: nasotracheal tube
O_2_
: oxygen
PaCO_2_
: arterial partial pressure of CO_2_ (mm Hg)
PaO_2_
: arterial partial pressure of O_2_ (mm Hg)
PEEP: positive end‐expiratory pressure (cmH_2_O)
PEF: peak expiratory flow (L/s)
PEP: peak expiratory pressure (cmH_2_O)
PIF: peak inspiratory flow (L/s)
PIP: peak inspiratory pressure (cmH_2_O)
Pmean: mean airway pressure (cmH_2_O)
Ppeak: peak airway pressure (cmH_2_O)
PS: pressure support (cmH_2_O)
PSV: pressure support ventilation
Rf: respiratory frequency (spirometry) (bpm)
RR: respiratory rate (observed) (bpm)
Te: expiratory time (s)
Ti: inspiratory time (s)
Vcap: volumetric capnography
V̇CO_2_
: CO_2_ production
Vd: dead space volume (L)
Vd_Bohr_
: dead space volume estimated using Bohr's method
Vt: tidal volume (mL)
Vt(e): expiratory tidal volume (mL)

## 1 INTRODUCTION

Respiratory disease is an important cause of morbidity and death in foals presented for veterinary care, associated with prematurity or dysmaturity, aspiration of meconium or milk, pneumonia or systemic infection.^1^ Because lung function is performance limiting even in healthy horses,^2^ and the majority of foals are intended for racing or other athletic pursuits, early and appropriate respiratory support is important to ensure optimal short and long‐term health outcomes.^3^

The administration of supplementary oxygen to foals is readily implemented but does not address respiratory insufficiency caused by inadequate ventilation, and is no longer considered optimal care for hypoxic human patients in some settings.^4^, ^5^ Positive pressure ventilation often is used in patients with respiratory insufficiency to improve gas exchange and decrease work of breathing. This approach is even more important in neonates given differences in lung physiology, pulmonary mechanics, and their lower metabolic reserves relative to adults. The use of commercial ventilators, designed for at‐home management of obstructive airway conditions in people, has identified improved gas exchange and mechanics of breathing in foals during noninvasive ventilation (NIV) using continuous positive airway pressure (CPAP)^6^ or bi‐level positive airway pressure (bi‐PAP).^7^ Although attenuated by bi‐PAP, modest hypercapnia was observed with both strategies, and improved characterization of the pulmonary effects of positive pressure ventilation modes in foals is needed.

In spontaneously breathing patients, respiratory support may be provided by CPAP or positive support ventilation (PSV). During CPAP, the ventilator maintains a positive airway pressure against which the patient must breathe.^8^ By contrast, PSV is a ventilation mode used when the rate or depth of ventilation is inadequate, where the clinician decides on a positive end‐expiratory pressure (PEEP) and a pressure over PEEP as peak inspiratory pressure (PIP).^8^ In both modalities, spontaneous respiration is preserved and ventilation is triggered by the patient.

Our study was designed to characterize the response of healthy foals with reversible respiratory insufficiency to CPAP or PSV delivered using a transport ventilator designed for use in humans, and to characterize the effects on respiratory mechanics of variable positive airway pressures for both ventilation modes. Based on previous reports we hypothesized that CPAP and PSV would be associated with improved oxygenation and respiratory mechanics, and that increased airway pressures would increase hypercapnia because of increased physiologic dead space.

## 2 MATERIALS AND METHODS

### 2.1 Research animals and experimental design

Six research foals (3 colts and 3 fillies) with a mean age of 23 days (range, 8‐29 days) and mean body weight of 73.5 kg (range, 65‐92 kg) were included in the study. All foals were owned by Charles Sturt University for undergraduate teaching and were of Connemara cross breeding (to Thoroughbred or Standardbred mares). Foals were free of respiratory or systemic disease at the time of recruitment and for the duration of the study, based on clinical examination, spirometry, and blood gas analysis. All foals were part of a concomitant study to ascertain effects of sedation, body position, and CPAP on distribution of ventilation using electrical impedance tomography.^9^ The study protocol was approved by the Charles Sturt University Animal Care and Ethics Committee (ACEC A19218) and by the Murdoch University Research Ethics and Integrity Office (N3190/19).

The study was conducted to assess the effects of CPAP and PSV during stepwise increases in inspiratory and expiratory pressures on respiratory mechanics assessed by spirometry and volumetric capnography (Vcap), pulmonary aeration assessed by computed tomography (CT) and gas exchange assessed by arterial blood gases (ABGs). Two treatment protocols (CPAP and PSV, Table 1) were allocated in a randomized crossover design with the first treatment assigned by coin toss for the 1st, 3rd, and 5th foals. The 2nd, 4th, and 6th foals received the alternative treatment protocol during the first phase of the study. Treatment settings were reversed for each foal in the 2nd phase of the study, and the interval between interventions ranged from 48 to 72 hours. Study design and sampling protocol are summarized in Table 2. Before each study, foals were manually restrained for veterinary examination and anaerobic collection of baseline ABG samples from the carotid artery (T‐1, Table 2). A 14G, 89‐mm catheter (Terumo Surflo, Macquarie Park, Australia) was placed in the right jugular vein of each foal and telemetric electrocardiography (ECG; Televet Telemetric ECG and Holter, Engel Engineering Service GmbH, Heusenstamm, Germany) was commenced for all foals using electrodes placed on the left side of the thorax. Spirometry and Vcap were performed, as described below at times shown in Table 2.

**TABLE 1:** Pressure settings allocated during continuous positive airway pressure (CPAP) and pressure support ventilation (PSV) in 6 healthy foals with reversible respiratory insufficiency.

Pressure settings (cmH_2_O)	CPAP			PSV		
	T1	T2	T3	T1	T2	T3
Positive end‐expiratory pressure	4	7	10	2	4	4
Pressure support	4	7	10	10	10	12
Peak inspiratory pressure	8	14	20	12	14	16

*Note*: Each intervention (T1, T2, and T3) lasted for 10 minutes.

**TABLE 2:** Sample schedule overview showing foal sedation and position, sample collection, and respiratory support.

Time	Activity	Monitoring	ABG	Spiro + Vcap	CT	
T‐1	Standing unsedated	HR‐RR‐T	✓	✓✓		
T0	Dorsal FiO_2_ 21%	HR‐RR‐T‐BP	✓	✓✓	✓	Insp breath hold at 5 cm H_2_O
T1 (CPAP 4/4 or PSV 10/2)	Dorsal FiO_2_ 21%	HR‐RR‐T‐BP	✓	✓✓	✓	Insp breath hold at PIP
T2 (CPAP 7/7 or PSV 10/4)	Dorsal FiO_2_ 21%	HR‐RR‐T‐BP	✓	✓✓	✓	Insp breath hold at PIP
T3 (CPAP 10/10 or PSV 12/4)	Dorsal FiO_2_ 21%	HR‐RR‐T‐BP	✓	✓✓	✓	Insp breath hold at PIP

*Note*: Continuous positive airway pressure (CPAP) or pressure support ventilation (PSV) was provided at T1, T2, and T3 with pressure support and positive end‐expiratory pressure (PS/PEEP) as shown.

Abbreviations: ABG, arterial blood gas; BP, blood pressure; CT, computed tomography; FiO_2_, O_2_ fraction in inspired air; HR, heart rate; insp, inspiratory; PIP, peak inspiratory pressure; RR, respiratory rate; spiro, spirometry; T, temperature; Vcap, volumetric capnography.

Foals were sedated with diazepam (0.2 mg/kg; Parnell Laboratories, Alexandria, Australia) 5 minutes before bolus injection of fentanyl (5 μg/kg; Hospira, Melbourne, Australia) and xylazine (0.2 mg/kg, Illium Veterinary Products, Glendenning, Australia), administered via the jugular catheter, and placed in right lateral recumbency. A continuous infusion of fentanyl (5 μg/kg/h), xylazine (0.7 mg/kg/h), and midazolam (0.1 mg/kg/h; Alphapharm Pty Ltd, Carole Park, Australia) in 0.9% sodium chloride delivered using syringe pumps (Alaris IMED Gemini PC‐1 infusion pump; VetQuip Pty Ltd, Erskine Park, NSW) was commenced and a cuffed nasotracheal tube (NTT) was placed. Foals then were placed on the CT table in dorsal recumbency, and a 22G, 2.5‐cm polyurethane catheter (Terumo Surflo, Macquarie Park, Australia) was placed in the left or right facial artery to facilitate arterial blood sampling. Standard monitoring equipment (noninvasive blood pressure monitoring and pulse oximetry) was placed, and a combined airway connector for capnography and spirometry measurements was placed between the NTT and y‐piece of the ventilator (NICO; Respironics, Wallingford, Connecticut). To avoid development of hypoxemia during instrumentation, supplementary oxygen was administered directly to the NTT via an oxygen port adaptor to achieve inspired oxygen concentration (FiO_2_) of 50%‐60%, confirmed by gas analysis using a Spectrum OR with Gas Module SE (Datascope Corporation, Mahwah, New Jersey).

Administration of supplementary O_2_ was discontinued after instrumentation, and foals remained with spontaneous respiration and FiO_2_ of 21% (room air) for an additional 10 minutes before sample collection and CT image acquisition (T0, Table 2). Respiratory support (as shown in Table 1) was commenced thereafter, with each intervention maintained for 10 minutes before sample collection and CT image acquisition, as described below.

Respiratory support was provided using a portable multipurpose ventilator (MTV1000 Dual Limb Ventilator System, MEK‐ICS Company Ltd, Gyeonggi‐do, Republic of Korea, distributed by Mediquip Pty Ltd, Loganholme, Queensland) using inspiratory and expiratory limbs and nonhumidified room air. Inhalation and exhalation flow trigger sensitivities for the ventilator to administer a pressure support breath were left at default values (30%). The ventilator switched from spontaneous ventilatory support during CPAP to PSV after apnea >20 seconds was detected. After each pressure change, the inspiratory and expiratory tidal volumes were examined and the filling of the cuff of the NTT readjusted if the difference between inspiratory and expiratory volume was >10% of the tidal volume. After collection of T3 samples and data, foals were returned to a mattress for recovery, the NTT and vascular catheters were removed, and the time to sternal recumbency and standing were recorded. Foals were re‐examined 24 hours after study completion and the replicate protocol was commenced after a minimum of 48 hours recovery.

### 2.2 Standard monitoring

Heart rate (HR) was determined by thoracic auscultation and by continuous telemetry ECG traces recorded for the duration of sample procurement. Respiratory rate (RR) was determined by observation of respiratory movements. Rectal temperature was recorded before each study and when samples were collected for blood gases determination. Indirect oscillometric arterial blood pressures were monitored (NIBP, Trimline Medical Products, Branchburg, New Jersey) during the final 2 minutes of each intervention (T0‐T3) using a cuff placed around the base of the tail. The mean blood pressure of 3 separate and consistent readings was recorded.

### 2.3 Blood gas analysis

The ABG samples were collected anaerobically from the carotid artery in standing foals and from the catheterized facial artery directly into heparinized syringes (BD heparinized syringes, BD, North Ryde, Australia). Catheters were irrigated with heparinized saline after sample collection, and dead space fluid was discarded before sampling. All samples were analyzed (GEM Premier, Model 3500; Abacus ALS, Macquarie Park, Australia) within 5 minutes of collection, or stored on ice and analyzed within 60 minutes (4 samples) to provide temperature‐corrected PaO_2_, oxygen saturation (sO_2_), PaCO_2_, and pH.

### 2.4 Spirometry and Vcap

Spirometry was performed on standing by application of a large veterinary anesthesia mask (SurgiVet large canine mask, product number 32393B1; Sound Veterinary Equipment, Rowville, Vic) placed on the foal's muzzle in such a way as to exclude air leaks and to minimize dead space but not prevent opening of the nares. The mask was attached to a connector designed for adult humans and containing a combined mainstream CO_2_ infrared and differential pressure sensor (NICO; Respironics, Wallingford, Connecticut). After placement of the NTT, spirometry was performed by attaching the sensor directly to the NTT. The capnograph was calibrated following manufacturer's guidelines before each experiment and the accuracy of the spirometer verified before and after commencing the study using a 500‐mL calibration syringe. Spirometry and Vcap data were recorded on a breath‐by‐breath basis on a laptop computer using dedicated software (Datacoll, Respironics, Wallingford, Connecticut).

Spirometry variables (tidal volume [Vt]; respiratory rate [Rf]; inspiratory and expiratory times [Ti and Te]; PIP; positive end‐expiratory pressure [PEEP]; peak inspiratory and peak expiratory air flows [PIF and PEF]) were determined by post‐sampling analysis of 6 consecutive and artifact‐free breath cycles representative of tidal breathing using dedicated software (FlowTool Viewer 2, September 10, 2005; Respironics, Wallingford, Connecticut). Volumetric capnography variables, including CO_2_ production per breath (V̇CO_2_), Bohr's physiological dead space^10^ (Vd_Bohr_), and end‐tidal CO_2_ (ETCO_2_) were taken directly from capnograph results. Enghoff's index (Index_Eng_) was calculated retrospectively from mixed expired CO_2_ and PaCO_2_ using the following equation: Index_Eng_ = (PaCO_2_ − PeCO_2_)/PaCO_2_.

### 2.5 Thoracic imaging

Thoracic CT images were obtained from all foals at T0, T1, T2, and T3 using a Toshiba Alexion 16‐Slice Helical CT scanner with a slice thickness of 1.0 mm and slice interval of 0.8 mm (1.0 × 0.8). Respiratory movements were arrested during image acquisition by application of positive airway pressure of 5 cmH_2_O for foals during T0 or to match PIP during CPAP or PSV.

Lung volumes were calculated by an imaging associate not involved in image acquisition and blinded to respiratory support protocols using dedicated software (Vitrea Enterprise Imaging version 6.6.2; Vital Images, Inc, Minnetonka, Minnesota). Mean attenuation values were determined after calculation of lung volume, and regions of interest (ROIs) were defined in dorsal lung fields using multiplanar reformatting. To ensure consistent evaluation, 6 1‐cm diameter ROIs were defined in 2 dorsal plane images for each foal, as shown in Figure S1. Dorsal plane images were evaluated at the level of the 9th, 11th, and 13th intercostal spaces, or at the level of the 7th, 9th, and 11th intercostal spaces if lung fields did not extend to the 13th rib. The first series was evaluated at the level of the dorsal aspect of the vertebral body (dorsal ROI) and the next series at the level of the ventral aspect of the vertebral body (ventral ROI), as viewed in the corresponding sagittal plane. Each ROI was placed midway between the vertebral body and the thoracic wall, axial to the ribs, with care taken to avoid blood vessels and bronchi. Hounsfield units in each of the 6 ROI were averaged to give a mean ROI value for each plane.

### 2.6 Statistical methods

Power analysis from previous studies^6^, ^7^ indicated that a sample size of 6 foals would discriminate differences in PaO_2_ and PaCO_2_ of 15 and 5 mmHg, respectively, with a power >0.80 and *α* = 0.05. A 2 treatment, 2 phase, cross‐over design was selected to increase statistical power and to control for individual differences and possible effects attributable to treatment order. Data were tested for normality using the Shapiro‐Wilks test and explored using appropriate descriptive statistics. Possible effects of replicate (1st or 2nd repeat) and sequence (CPAP/PSV as first treatment) were evaluated by fitting separate mixed effects models using restricted maximum likelihood (REML) with time and replicate or sequence as random factors and foal as a fixed factor. In the absence of significant replicate or sequence effects, treatment effects (CPAP/PSV) were determined by comparison of responses during ventilation (T1, T2, and T3) against results in dorsal recumbency breathing room air (T0) by mixed effects models with time and treatment as random factors, subject (foal) as a fixed factor and post hoc testing by Tukey's method. The Greenhouse‐Geisser correction was used to control for unequal sphericity. Correlations between measured spirometry and ABG variables were explored by univariable analysis using Pearson coefficient. Significance was accepted as *P* < .05 and all analyses were performed using Graph Pad Prism 8.4.3 for Windows (GraphPad Software, San Diego, California, www.graphpad.com).

## 3 RESULTS

Foals tolerated all interventions well, and no adverse effects were observed for the duration of the study. No replicate or sequence effects were observed for any study parameter. Heart rate, RR, and rectal temperature decreased throughout the study intervention (all *P* < .001, Figure S2), but no time effect was observed for blood pressure parameters, and no difference was found between CPAP and PSV protocols for any vital parameter.

### 3.1 Spirometry and Vcap

**FIGURE 1:**
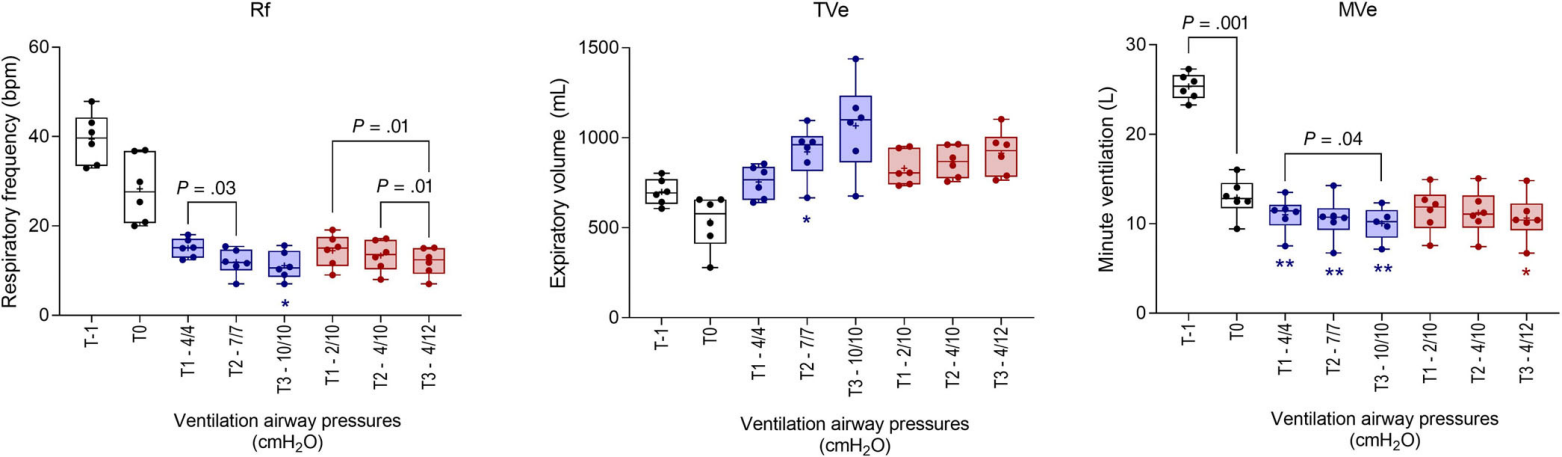
Effects of continuous positive airway pressure and pressure support ventilation protocols on respiratory rate (Rf), tidal volume (TVe), and minute ventilation (MVe) at T1, T2, and T3 compared with results from sedated foals in dorsal recumbency without ventilatory support (T0; *, *P* < .05; **, *P* < .01). Significant differences within different ventilation protocols are shown. Results at T‐1 and T0 are the mean result for each foal derived from both phases of the study. Results are shown as median, mean (+), quartiles (box), and range (whiskers), with all data points included.

**FIGURE 2:**
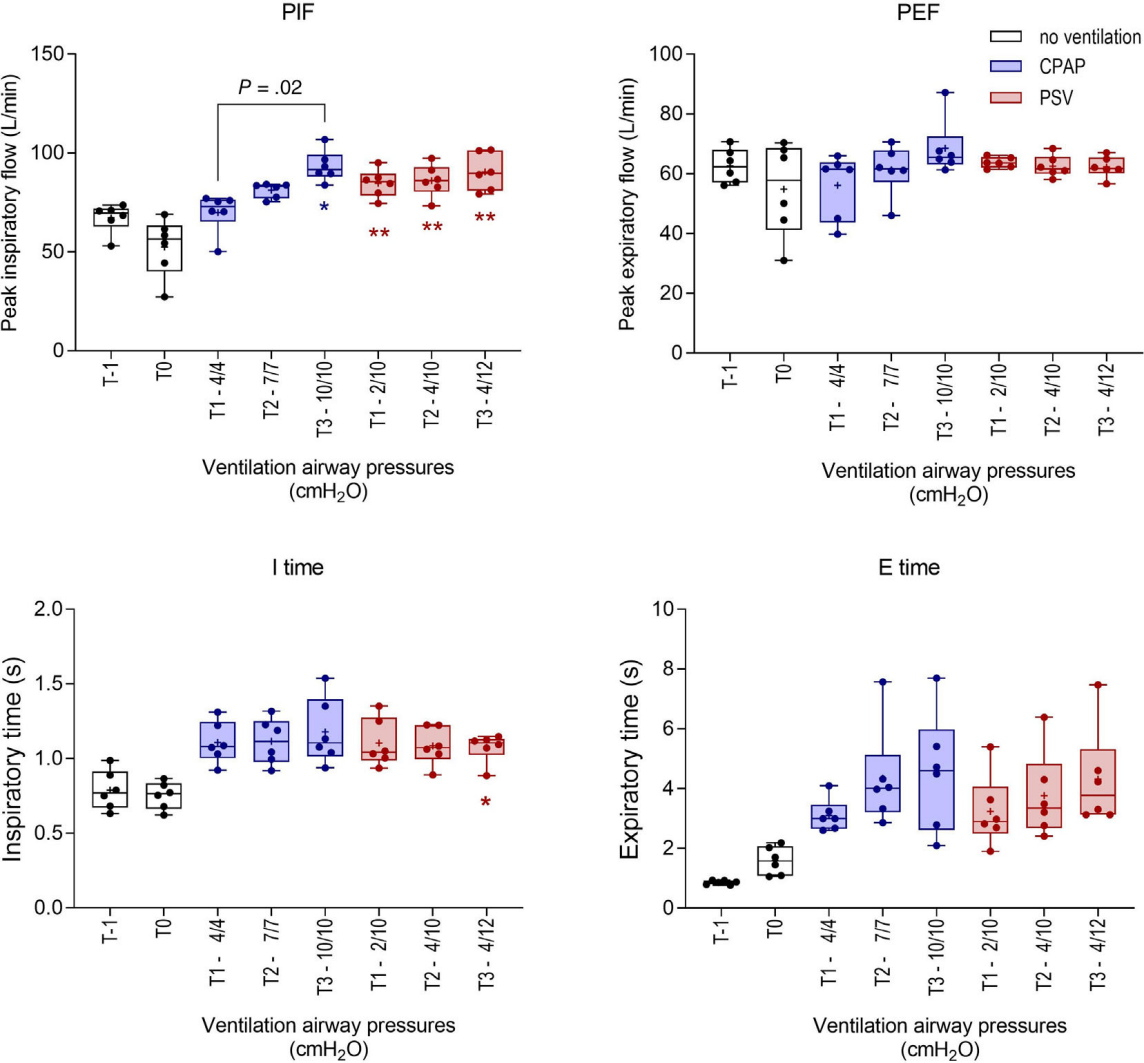
Effects of continuous positive airway pressure (CPAP) and pressure support ventilation (PSV) protocols on peak inspiratory and expiratory flows (PIF and PEF), inspiratory and expiratory times (Ti and Te) at T1, T2, and T3 compared with results from sedated foals in dorsal recumbency without ventilatory support (T0; *, *P* < .05; **, *P* < .01). Significant differences within different ventilation protocols are shown. Results at T‐1 and T0 are the mean result for each foal derived from both phases of the study. Results are shown as median, mean (+), quartiles (box), and range (whiskers), with all data points included.

**FIGURE 3:**
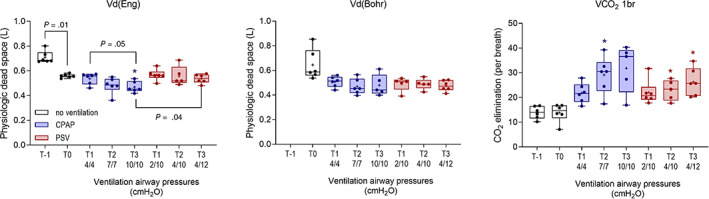
Effects of continuous positive airway pressure (CPAP) and pressure support ventilation (PSV) protocols on selected capnography variables at T11, T22, and T33 compared with results from sedated foals in dorsal recumbency without ventilatory support (T0; *, *P* < .05; **, *P* < .01). Significant differences within and between different ventilation protocols are shown. Results at T‐1 and T0 are the mean result for each foal derived from both phases of the study. Results are shown as median, mean (+), quartiles (box), and range (whiskers), with all data points included.

Changes in spirometry variables are shown in Figures 1 and 2. As noted for RR, Rf was decreased in sedated foals, and was further decreased during ventilation (*P* < .001), with a significant difference, relative to T0, observed during CPAP at T3. Significant decreases in Rf, relative to T0, were present in both ventilation protocols (Figure 1). All foals during CPAP 10/10, and 1 foal during CPAP 7/7 experienced periods of apnea >20 seconds, and the ventilator switched to PSV. Tidal volume (TVe) increased (*P* = .001) during ventilation, but observed differences, relative to T0, were significant only during CPAP at T2. Minute ventilation decreased marginally but significantly during CPAP and PSV 4/12 (*P* < .001; Figure 1). Peak inspiratory flow (PIF) increased significantly as compared to unventilated foals in dorsal recumbency (T0) associated with both CPAP and PSV protocols (*P* < .001; Figure 2), with no difference evident between respiratory support protocols and no effect observed on peak expiratory flows (PEF). Consistent with observed changes in Rf, increased Ti and Te were observed during both ventilation modes (both *P* = .003; Figure 2), but differences in comparison with nonventilated, sedated foals in dorsal recumbency (T0) were significant only for Ti during PSV at T3. Ventilation had a significant effect on Index_Eng_ and V̇CO_2_ elimination (*P* < .001 and *P* = .004, respectively). Enghoff's index decreased significantly at T3 during CPAP (Figure 3). Increased V̇CO_2_ elimination per breath was evident in both ventilation modes (Figure 3). Bohr's physiologic dead space (Figure 3) and end‐tidal CO_2_ (not shown) did not change during CPAP or PSV.

### 3.2 Arterial blood gas results

**FIGURE 4:**
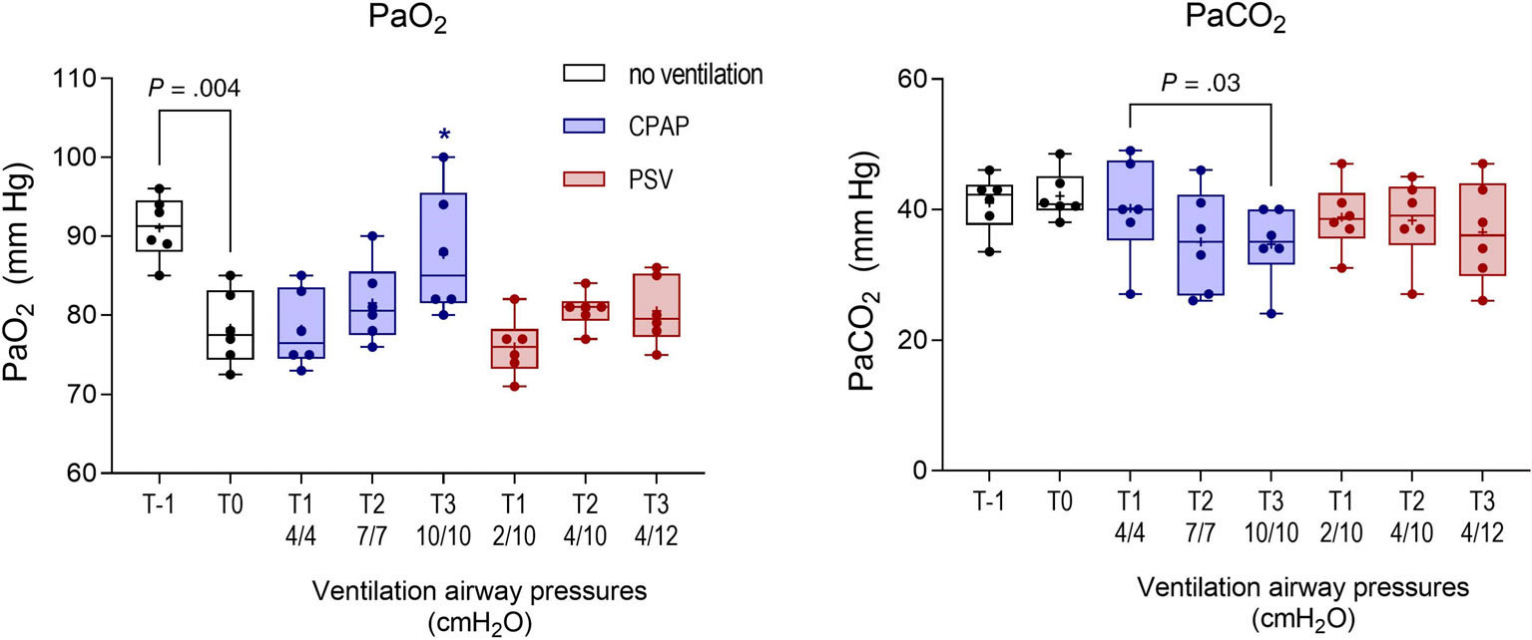
Effects of continuous positive airway pressure (CPAP) and pressure support ventilation (PSV) protocols on arterial blood gas results at T11, T22, and T33 compared with results from sedated foals in dorsal recumbency without respiratory support (T0). A significant effect on PaO_2_ was evident at T5 for foals receiving CPAP (*P* = .03) relative to values obtained for foals in dorsal recumbency at T0, as shown (*, *P* < .05). There was no effect on PaCO_2_. Results at T‐1 and T0 are the mean result for each foal derived from both phases of the study. Results are shown as median, mean (+), quartiles (box), and range (whiskers), with all data points included.

Mean PaO_2_ in standing foals (T‐1) was 90.7 mm Hg (range, 85‐96 mm Hg). The PaO_2_ varied significantly with time (*P* = .005; Figure 4), with a significant decrease evident in nonventilated sedated foals in dorsal recumbency (T0) relative to standing, unsedated foals (T‐1). Administration of CPAP was associated with increased PaO_2_, and a significant effect was observed at T3 as compared to results for foals in dorsal recumbency without ventilation (T0, Figure 3). Values observed at this time were not significantly different from results obtained in standing foals (T‐1). During both ventilation modes, PaO_2_ was positively correlated with mean airway pressure (*r* ^2^ 0.450, *P* < .001), PIP (*r* ^2^ 0.421, *P* < .001), and PEEP (*r* ^2^ 0.211, *P* = .005) determined by spirometry, and with ventilator settings for PEEP (*r* ^2^ 0.4346, *P* < .001) and PIP (*r* ^2^ 0.271, *P* = .001).

Mean PaCO_2_ in standing unsedated foals was 41.0 mm Hg (range, 33.5‐46.0 mm Hg). No effect of ventilation was observed on PaCO_2_, relative to that seen in dorsally recumbent foals (Figure 4). No difference in PaCO_2_ was observed within or between treatment groups, and ventilation mode was not associated with an effect on PaCO_2_. Values obtained for PaCO_2_ were weakly but positively correlated with PIF (*r* ^2^ 0.163, *P* = .01), expiratory time (Te, *r* ^2^ 0.161, *P* = .01), and mixed expired CO_2_ (PeCO_2_, *r* ^2^ 0.131, *P* = .03).

### 3.3 Thoracic imaging

**FIGURE 5:**
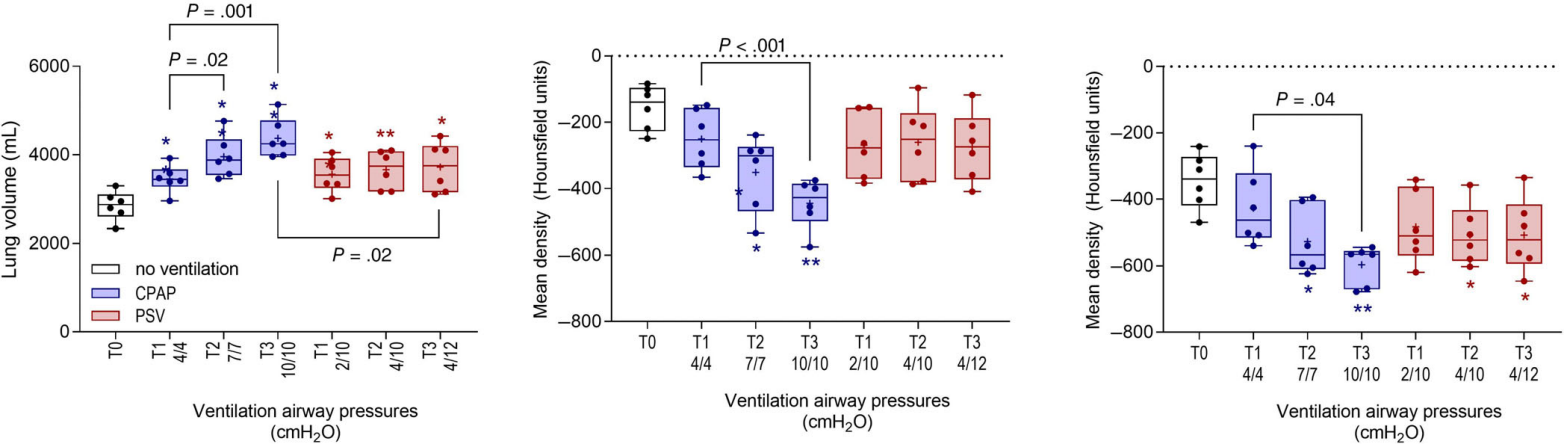
Effects of continuous positive airway pressure (CPAP) and pressure support ventilation (PSV) protocols lung volume (left) and attenuation in regions of interest in dorsal (middle) and ventral (right) regions of interest at T1, T2, and T3 compared with results from sedated foals in dorsal recumbency without respiratory support (T0). Significant differences within and between treatments are shown; differences in comparison with results obtained at T0, before ventilation, are also shown (*, *P* < .05; **, *P* < .01). Results at T‐1 and T0 are the mean result for each foal derived from both phases of the study. Results are shown as median, mean (+), quartiles (box), and range (whiskers), with all data points included.

**FIGURE 6:**
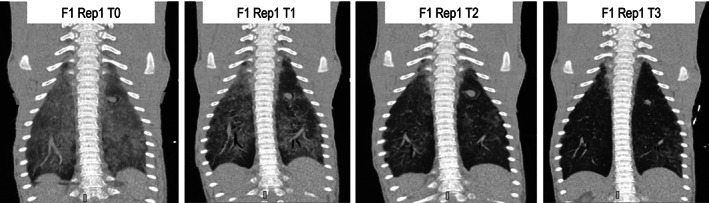
Representative dorsal plane computed tomography images from Foal 1 at T0 (left), T1, T2, and T3 during the continuous positive airway pressure protocol.

Lung volumes were lowest in unventilated foals at T0 (mean, 2798 mL; range, 2174‐3382 mL). Significant and incremental effects of ventilation were observed on lung volume and attenuation in ventral ROI associated with both protocols (Figure 5). Lung volume increased relative to values observed at T0 during both CPAP and PSV protocols, and a significant difference in density was evident during both protocols in ventral lung fields and in dorsal lung fields during CPAP (Figure 5). Attenuation of lung fields was subjectively and progressively decreased on subjective evaluation of sequential images (Figure 6). Lung volume was positively associated with measured expiratory and inspiratory volumes (*r* ^2^ 0.515, *P* < .001, and *r* ^2^ 0.485, *P* < .001, respectively), Pmean (*r* ^2^ 0.375, *P* < .001), PIP (*r* ^2^ 0.357, *P* < .001), inspiratory time (Ti, *r* ^2^ 0.309, *P* < .001), Te (*r* ^2^ 0. 227, *P* = .003), PEEP (*r* ^2^ 0.199, *P* = .006), and V̇CO_2_ per breath (*r* ^2^ 0.471, *P* < .001), and with ventilator settings for PEEP (*r* ^2^ 0.320, *P* < .001) and PIP (*r* ^2^ 0.277, *P* = .001).

## 4 DISCUSSION

Ours is the first study comparing 2 positive pressure ventilation modes in foals. Both CPAP and PSV were well tolerated by sedated healthy foals, and both modes improved gas exchange with stable ventilation in dorsal recumbency as evidenced by increased PaO_2_ and V̇CO_2_, and stable PaCO_2_. At the airway pressures used, Index_Eng_ as a global indicator for ventilation/perfusion (V/Q) mismatch^11^, ^12^ decreased and VD_Bohr_ remained stable, allowing the conclusion that V/Q matching was improved without causing overdistension of the lungs.

In our study, CPAP was associated with improved arterial oxygen pressures, an effect that was most strongly correlated with mean airway pressure during ventilation. This outcome is attributable to enhanced gas exchange associated with improved V/Q matching. Contrary to our hypothesis, higher airway pressure values were not associated with hypercapnia or increased CO_2_ levels. This observation, together with the finding that minute ventilation was unchanged, supports the conclusion that, at the airway pressures used, no overdistension of healthy lung tissue occurred. Overdistension would increase alveolar dead space and therefore cause increased PaCO_2_ at constant minute ventilation.^12^ Again, this conclusion was confirmed by Vcap findings because no change in Vd_Bohr_ occurred. Bohr's physiological dead space is the sum of airway and alveolar dead space and therefore the part of the tidal volume that does not participate in gas exchange.^13^ Based on earlier reports^6^, ^7^ we hypothesized that physiological dead space and PaCO_2_ would increase as alveolar dead space was increased with higher airway pressures associated with alveolar overdistension and consequently decreased perfusion. However, our hypothesis was not confirmed because no increase in PaCO_2_ or increase in physiologic dead space was observed. This finding shows that PIPs up to 20 cmH_2_O can be used in healthy foals without negative impact on dead space ventilation.

The observed increased elimination of CO_2_ with increased airway pressures has been reported previously,^6^, ^7^ and further indicates that ventilation was improved in our study. Furthermore, the stable or improved CO_2_ elimination supports the assumption that the applied airway pressures had no clinically relevant effect on cardiac output, because V̇CO_2_ is influenced by changes in ventilation or lung perfusion, and the later directly depends on cardiac output.^14^

As previously reported,^6^, ^7^ CPAP and PSV were well tolerated in healthy sedated foals in our study, with no adverse effects observed on HR or blood pressure. The effects of the 2 ventilation modes were well visualized using CT, where increased pulmonary volume and decreased attenuation in dependent lung fields were evident. The findings are consistent with expected benefits of positive airway pressure including recruitment of collapsed alveoli and an increased pulmonary exchange membrane. Thoracic CT imaging has been used to assess lung volume^15^, ^16^ and pulmonary pathology in foals,^17^, ^18^ and effectively identified improved aeration associated with ventilation in our study.

As in previous studies of CPAP and biPAP in foals, spirometry identified decreased RR and increased tidal volumes during ventilation in our study, and this finding is recognized as a more efficient ventilation strategy. Together with the observed increase in PIF, these findings are consistent with improved respiratory mechanics or decreased work of breathing. The tidal volume in our study during CPAP and PSV ranged from 8.8 to 18.9 mL/kg and 8.7 to 14.3 mL/kg, respectively.

During PSV and CPAP, periods of apnea were common, especially during CPAP of 10 cmH_2_O, where the ventilator switched to PSV. Apnea periods might be attributed to activation of the Hering‐Breuer reflex in response to increased end expiratory pressure, or associated with direct activation of airway stretch receptors. The transport ventilator used in our study was able to initiate a breath in the event that patient‐initiated inspiration was delayed, as was the case for “off‐the‐shelf” devices used previously. In contrast to previous studies, the transport ventilator used in our study allowed longer expiratory times, with capacity to set an acceptable period of apnea (from 20 to 60 seconds).

Changes in venous return might be expected with increased intrathoracic pressure, which could decrease cardiac output, but this situation has not been observed in our studies to date. Our current study was an interventional study on a small number of healthy foals with pharmacologically induced respiratory insufficiency. Study findings therefore could be cautiously applied to the management of neonates with spontaneous respiratory disease, and failure to recruit foals with spontaneous respiratory disease is a study limitation. Patient responses to ventilation may be unpredictable and are likely to be labile. Individual patients may have variable recruitable lung volumes on presentation, and might be expected to respond differently over time during treatment and to both ventilation modes used in our study.^19^, ^20^ As in human patients, the underlying pathophysiology of respiratory insufficiency in foals is dynamic, and changes in respiratory drive may require frequent manipulation of ventilator settings, particularly if the patient is at an unstable point in the disease process.^21^ Our study, however, gives the clinician important basic guidelines on ventilator settings that are well tolerated by healthy lung tissue and especially is of importance because 2 ventilation modes were compared. The clinician can select a mode where foals are supported in their spontaneous ventilation during CPAP or, if foals are more fatigued, PSV settings are described where each breath is supported by the ventilator. Furthermore, both ventilation modes can be used during NIV using an interface between ventilator and patient that does not require intubation of the trachea, as has been reported previously.^6^, ^7^

In our study, ABG, spirometry, Vcap, and CT were used to characterize pulmonary responses to ventilation. However, ABG sampling and CT are invasive, intermittent, and may not be available in a clinical setting. Spirometry and Vcap are used to monitor human patients during ventilation, but their accuracy may be limited during NIV because of leakage associated with patient‐machine interfaces. There is a need, therefore, for improved measures of respiratory function to detect respiratory compromise in foals and to monitor patient response. Foals in our study were intubated to facilitate breath‐hold during thoracic CT image acquisition and to exclude leakage during ventilation, but this approach would not be desirable during NIV in a clinical setting. Additional studies to refine patient‐ventilator interfaces to decrease leak and enable accurate monitoring are required.

## 5 CONCLUSIONS

Our study determined that ventilation using a transport ventilator designed for use in humans was associated with increased PaO_2_ during CPAP and PSV, with no adverse effect on PaCO_2_. Together with a decrease in Index_Eng_, increased CO_2_ elimination, and no change in Vd_Bohr_, these findings support the conclusion that the airway pressures used improved V/Q matching without overdistension of lung tissue of healthy foals. Ventilation was associated with decreased respiratory rate (RR and Rf) and slightly decreased minute ventilation, relative to results obtained during unsupported respiration. All results reflect more efficient ventilation and decreased work of breathing during CPAP and PSV. These effects were more pronounced during CPAP, and were not observed associated with corresponding PSV values when lower PEEP and PIP levels were used. Additional studies are required to determine optimal pressure support and PEEP strategies for foals with respiratory compromise. Improved methods of noninvasive assessment of respiratory function are likely to facilitate improved respiratory support of equine neonates.

## CONFLICT OF INTEREST DECLARATION

Authors declare no conflict of interest.

## OFF‐LABEL ANTIMICROBIAL DECLARATION

Authors declare no off‐label use of antimicrobials.

## INSTITUTIONAL ANIMAL CARE AND USE COMMITTEE (IACUC) OR OTHER APPROVAL DECLARATION

Approved by the Charles Sturt University Animal Care and Ethics Committee (ACEC A19218) and by the Murdoch University Research Ethics and Integrity Office (N3190/19).

## HUMAN ETHICS APPROVAL DECLARATION

Authors declare human ethics approval was not needed for this study.

## Supporting information


**Data S1.** Supporting Information.
