# Supplementary material for: Effects of 2 modes of positive pressure ventilation on respiratory mechanics and gas exchange in foals
Source: J Vet Intern Med. 2023 Apr 13;37(3):1233–42. doi: 10.1111/jvim.16651 (PMC10229351; doi:10.1111/jvim.16651)

Supplementary Figure 1: Method used to define regions of interest for assessment of attenuation in the dorsocaudal lung fields. Regions of interest (ROI) were identified in two planes in the dorsal lung fields: one at the level of the dorsal aspect of the vertebral bodies, as shown (dorsal ROI), and one at the level of the ventral aspect of the vertebral bodies (ventral ROI, not shown). For both planes, mean attenuation was measures in each of six circular, 1cm diameter, ROI defined a located mid-way between the rib and the vertebrae at intercostal spaces (ICS) 9, 11 and 13 (or ICS 7, 9 and 11 if pulmonary tissue was not visible past the 13th rib).

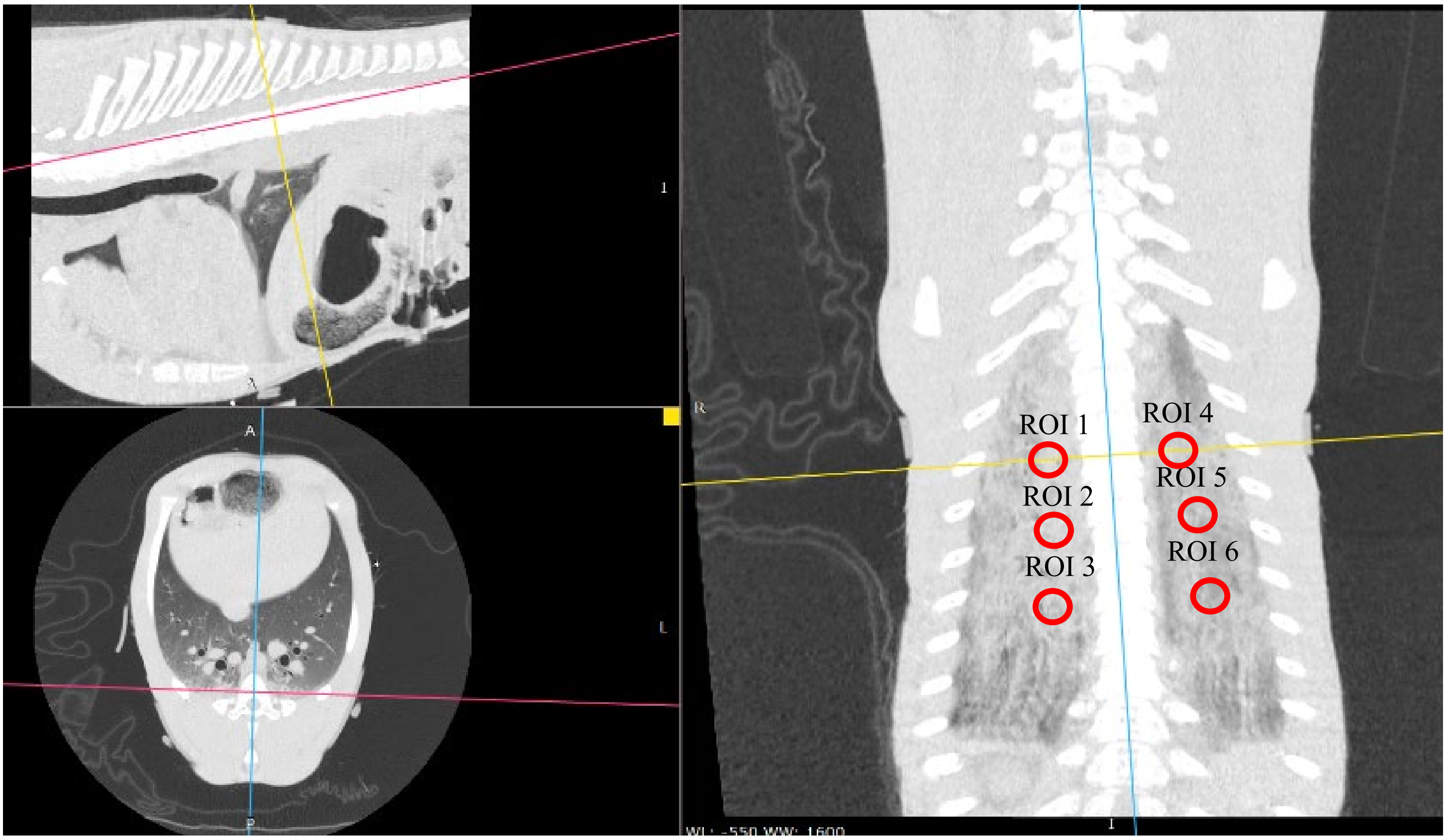

Supplementary Figure S2: Effects of high and low positive end expiratory pressure (PEEP) protocols on heart rate (HR), respiratory rate (RR), rectal temperature (temp), mean arterial pressure (MAP), systolic and diastolic blood pressure (sysBP, diaBP). Time had a significant effect (P≤0.001) on HR, RR and temp, but no effect on blood pressure parameters, and time:treatment interactions were not significant. Significant differences due to non-invasive ventilation within groups, in comparison with results observed at T2(air), are shown (*, P<0.05; **, P<0.005). Results are presented as mean ± 95% CI.

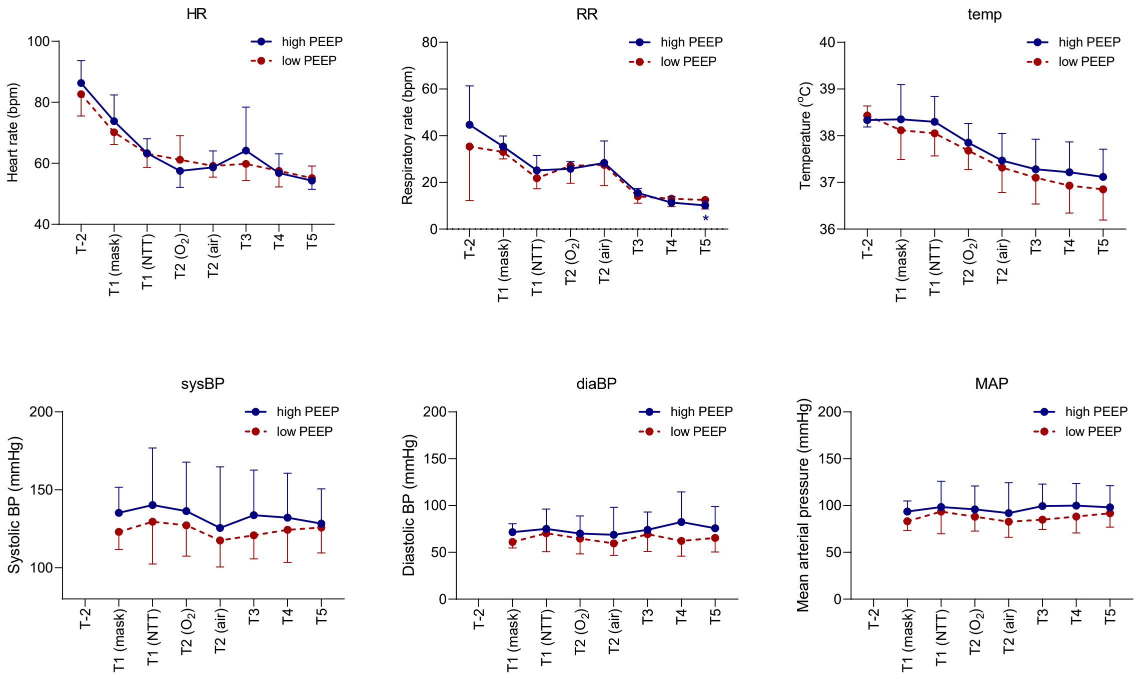

Supplementary Figure S3: Effects of high and low positive end expiratory pressure (PEEP) protocols on PaO_2 and $PaCO_2$.

Significant effects of time were observed for PaO_2 ($P<0.001$), but time-treatment interactions were not significant, and no significant differences were observed between high and low PEEP protocols at T3, T4 or T5. The administration of supplementary oxygen (T2-O2) was associated with significantly increased PaO_2 compared with results obtained at T1 (mask) and at T2 (air) (P=0.013 and P=0.007, respectively), and results for recumbent foals at T1 (mask) and T2 (air), were less than values observed in standing, unsedated foals at T-2 (P=0.006 and $P<0.001$, respectively). During NIV, and within the high PEEP group, PaO_2 was significantly increased at T5, relative to results obtained from dorsally recumbent, spontaneously breathing foals (T2-air, P=0.017, as shown); no significant differences were observed for foals receiving the low PEEP protocol. Within groups, differences during NIV, compared to results in standing, unsedated foals (T-2) are shown (*, $P<0.05$; ** $P<0.001$).

Time also had a significant effect on $PaCO_2$ (P=0.004), but time-treatment interactions were not significant and no significant effects were observed within either treatment group. Across both groups, $PaCO_2$ during the administration of supplementary oxygen T2 (O_2) was significantly less than observed at T1 (mask) or T3 (P=0.013 and P=0.019, respectively).

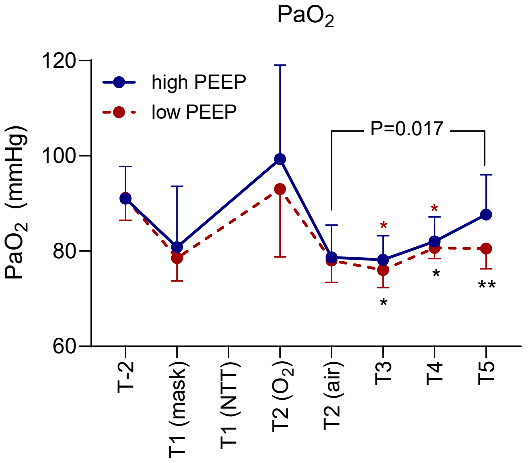

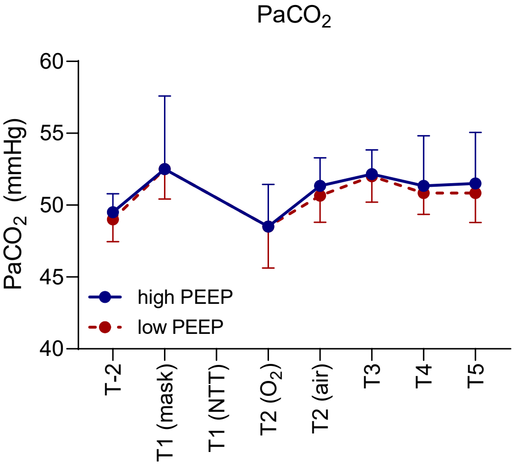

Supplementary Figure S4: Effects of high and low positive end expiratory pressure (PEEP) protocols on spirometry variables. Time had a significant effect (P<0.001) on all parameters except peak expiratory flow (P=0.139); time:treatment interactions were not significant. Significant differences due to non-invasive ventilation within groups, in comparison with results observed at T2(air), are shown (*, P<0.05; **, P<0.005). Results are presented as mean ± 95% CI.

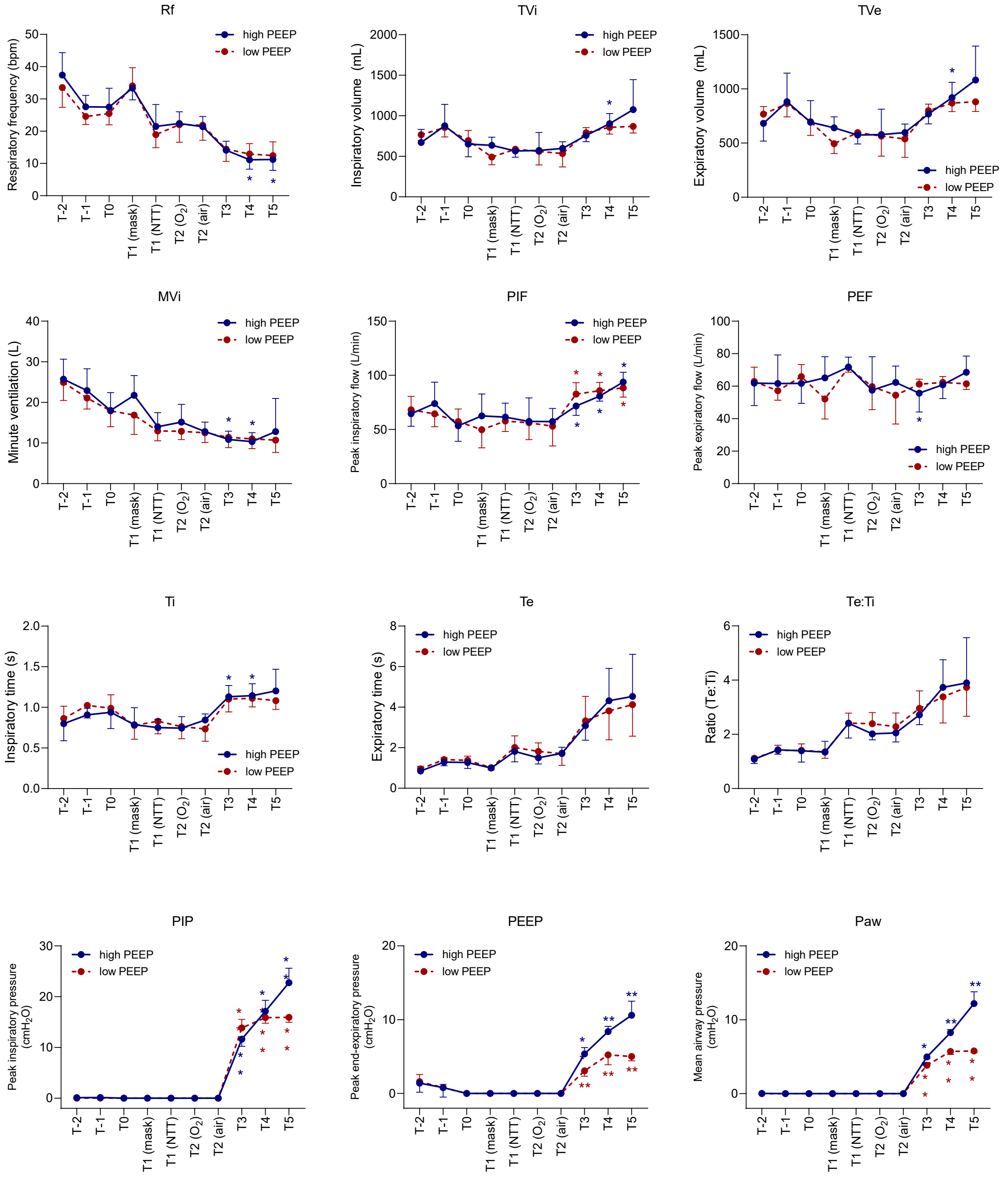

Supplementary Figure S5: Effects of high and low positive end expiratory pressure (PEEP) protocols on volume capnography variables. Time had a significant effect ($P<0.001$) on all parameters, but time:treatment interactions were not significant. Significant differences due to non-invasive ventilation within groups, in comparison with results observed at T2(air), are shown (*, $P<0.05$; **, $P<0.005$). Results are presented as mean ± 95% CI.

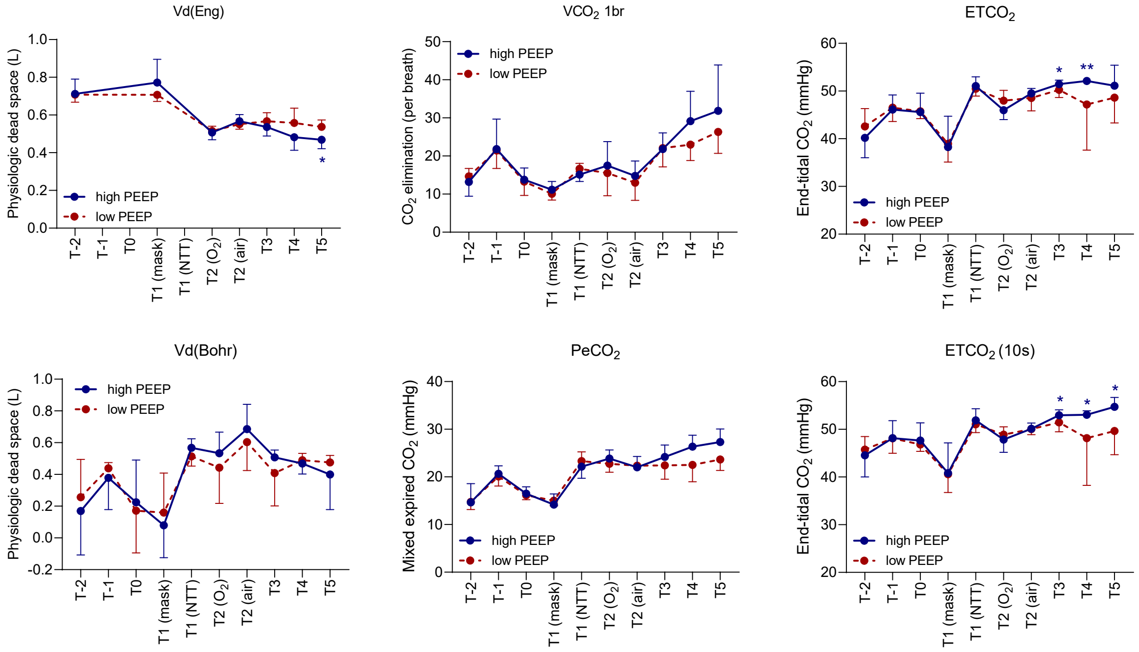

Supplement: Supplementary file 1 — Data S1. Supporting Information. [file JVIM-37-1233-s001.pdf]
